# Ethnicity estimation using family naming practices

**DOI:** 10.1371/journal.pone.0201774

**Published:** 2018-08-09

**Authors:** Jens Kandt, Paul A. Longley

**Affiliations:** 1 Centre for Advanced Spatial Analysis, University College London, London, United Kingdom; 2 Department of Geography, University College London, London, United Kingdom; Universitat Pompeu Fabra, SPAIN

## Abstract

This paper examines the association between given and family names and self-ascribed ethnicity as classified by the 2011 Census of Population for England and Wales. Using Census data in an innovative way under the new Office for National Statistics (ONS) Secure Research Service (SRS; previously the ONS Virtual Microdata Laboratory, VML), we investigate how bearers of a full range of given and family names assigned themselves to 2011 Census categories, using a names classification tool previously described in this journal. Based on these results, we develop a follow-up ethnicity estimation tool and describe how the tool may be used to observe changing relations between naming practices and ethnic identities as a facet of social integration and cosmopolitanism in an increasingly diverse society.

## 1. Introduction: Names and ethnic identity

Previous research has demonstrated that a name very often provides an informative marker of ethnic, linguistic and cultural origin [1–4] both on a global scale as well as within countries such as Great Britain [5–6]. Some of this research has been reported in this journal [7], wherein automated clustering procedures are used to detect ‘naming networks’ from directories representing the populations of 17 countries, provide a valuable representation of cultural, ethnic and linguistic population structure around the world. Such methods can be used to demonstrate how classes emerge from millions of individual parental naming decisions, but do not provide any evidence that the bearers of the resulting names themselves identify with the groups so identified.

In practice, names are defined, changed or adopted by humans at various moments in history and in the life course, they result inevitably from social naming practices, which occur within specific ethnic, cultural, spatial and secular contexts. In many societies, surnames are passed along patrilineal lines while forenames are chosen by parents according to prevailing local, cultural and temporal preferences [3, 6, 8, 9]. As a result, names can be an indicator of not only ethnic background but also gender and age.

Names are widely collected in almost every micro dataset including administrative registers, consumer data or social surveys. While in most datasets, names remain confidential data items that are not disclosed, ethnic affiliation is only collected as part of special purpose-built applications. Yet, ethnicity is an important component in many contemporary social debates, including in inequality of health outcomes and life chances, identity, social integration and segregation (e.g. [10–14]). Hence, names-based classifications offer significant utility in generating information on ethnicity while avoiding disclosure of person-identifiable data.

One limitation of names-based ethnic classifications is that they do not necessarily correspond to an individual’s subjective identification with an ethnic group, especially in contexts of increased ethnic mixing and integration of successive generations of initial immigrants. This discord between names-based and subjective attribution may be entirely desirable in some applications, such as those focussing on genetic or biological traits of ethnicity (e.g. [15]). In such studies, self-ascription may confound attribution of ethnicity, and therefore a classification that is more grounded in ancestral naming practices is preferred. But in studies focussing on social phenomena such as segregation or social integration, subjective identification becomes arguably more significant than the ancestral signal of names.

In this paper, we compare a names-based classification with self-ascribed ethnicity for the entire population in England and Wales in order to gauge the extent to which names-based classifications can be used in applications that deal with issues of subjective identities. The procedures we suggest as part of this comparison comprise an evaluation of Onomap [7], a widely used names-based classification generated from unsupervised machine-learning algorithms. Such evaluations are largely wanted in social science research on names. Based on specific recommendations arising from this process, we discuss the potential of names classifications to reflect subjective ethnic affiliations in England and Wales, and present a freely accessible tool to assist a range of applications.

## 2. Current classification of names

The facility to classify ethnicity is of increasing importance for statistical purposes because of continued international immigration, greater volatility of population movements over time and the limited capacity of traditional government surveys to measure such changes at appropriate spatial and temporal levels of detail. A number of names-based classifications of ethnicity have been developed in the past, including the widely-accessed Onomap tool [7], Nan Pechan [16], and the commercial OriginsInfo software (Webber-Phillips, London, UK: webberphillips.com/). Such classifications often process address registers (such as the public version of the UK Electoral Roll) or telephone directories and model pairwise associations of given and family names to develop clusters of names by means of unsupervised statistical learning procedures. Researchers subsequently label these clusters with reference to distinctive cultural, ethnic or linguistic groups. Alternatively, supervised learning procedures involving fuzzy matching techniques on labelled, historical datasets have also been successfully applied, offering valuable insights for specific research problems [17].

The Onomap classification (OM), which we focus on in this paper, results from an unsupervised classification procedure and does not have a built-in fuzzy matching component. It was developed using a 2007 list of adult (age 17+) names for the UK procured from CACI Ltd (London, UK). As most names-based classifications, OM does not reflect any form of subjective ethnic identification. Yet, the tools have been used in applications where these are likely at play, and we are not aware of any extensive studies that evaluate these classifications at the level of the individual. There are a number of further shortcomings to these classifications:

1. The data sources underlying the classifications provide incomplete and probably biased representations of the population-at-large. For example, public electoral registers do not include (young and immigrant) non-voters or (privacy-sensitive) ‘opt out’ individuals, and public telephone directories provide less than universal coverage and few given names.
2. Data sources are typically restricted to the 16+ age cohorts. They may be supplemented by lists of baby given names (such as those made available by statistical agencies in the UK and USA) but these lists do not accommodate names of the children who migrated with their parents. This makes it difficult to analyse a snapshot of the population at any specific moment in time.
3. There is no framework for accommodating individual self-assignments of ethnicity with modelled results. This is pertinent when, for example, seeking to identify individuals in the UK that might self-identify as ‘White Irish’.
4. Little focus has been developed upon groups whose names manifest multiple cultural origins, such as Caribbeans, whose ethnicity can likely only be ascertained through subtle associations between forename-surname pairings.
5. There has been no consideration of geographic context, such as national or regional naming patterns within the UK.

In order to address these concerns, this work—unlike existing classifications—puts the validation of ethnicity estimates at the heart of the modelling process to produce an expanded and updated dictionary of names. Unlike algorithmic classifications, this is informed by stated self-identifications of ethnicity, which may be plausibly linked to social and cultural affiliations. As such, the results of our analysis provide a valuable assessment of the limitations of unsupervised machine-learning solutions.

## 3. Evaluating and advancing names-based ethnic classifications

We use a new and novel method of data access to records of the 2011 UK Census of Population for England and Wales. Under the ‘100-year rule’, individual UK Census records will remain strictly confidential until 2111 and prior to that date only special, secure and indirect access for research purposes to these data may be negotiated through the UK Office for National Statistics (ONS) Secure Research Service (https://www.ons.gov.uk/aboutus/whatwedo/paidservices/virtualmicrodatalaboratoryvml). These secure arrangements enable us to access aggregate statistics derived through the application of algorithms applied to individual names recorded in the Census without any form of disclosure to the research. ONS staff run specified queries on these names and make aggregate, non-identifiable results available in a secure facility subject to suppression of small counts and disclosure control. Thus, the research team do not have access to individual level Census data. In addition, the research was subject to full ethical scrutiny by the National Statisticians Data Ethics Advisory Committee (NSDEC, https://www.statisticsauthority.gov.uk/about-the-authority/committees/nsdec/). The queries provide an evaluation of Onomap based on more recent data for England and Wales and thus present an improved method of predicting the most likely Census category to which individuals would assign themselves.

### 3.1. Census record classification and VML input

The Onomap (OM) classification arose from work conducted at University College London, reported in Mateos et al. [7], which demonstrates how ‘naming networks’, constructed from a large sample of forename-surname pairs provide a valuable representation of cultural, ethnic and linguistic population structure around the world. OM identifies clear social and ethno-cultural clusters in such naming networks that extend far beyond the geographic areas in which particular names originated, and that are preserved even after. This innovative approach enriches and adds value to automated population classification through conventional national data sources such as telephone directories and electoral registers.

OM reads new lists of names, matches them with its internal lookup table and assigns the ethnic group that is stored for each name. If ethnic groups differ between forename and surnames, the group of the surname is assigned. We requested ONS to run OM to classify all 51 million Census micro-records in England and Wales based on personal names where they had been captured with sufficient accuracy. ONS included every full, personal name (fore- and surname) with counts of more than 30 and compared the OM-predicted ethnic group with the self-assigned group of the bearer recorded in the Census, where this had been reported and not imputed. OM includes 185 ethno-cultural categories; and these were aggregated to the following 11 aggregate groups used in the 2011 Census:

- White: (1) White British, (2) White Irish, (3) Any Other White Background
- Asian: (4) Indian, (5) Pakistani, (6) Bangladeshi, (7) Chinese, (8) Any Other Asian Background;
- Black: (9) African, (10) Caribbean;
- Other: (11) Any Other Ethnic Groups, which is an aggregation of infrequently occurring Census categories of Arab, Mixed Background or Any Other Ethnic Group.

These very broad Census categories are aggregations that are deemed the most relevant by UK stakeholders and other users following extensive consultations [18]. The published results are thus a categorisation of groups that do not bear a direct correspondence with subjective, ethnic identity, although some may be more granular when referring to common immigrant groups in the UK (e.g. ‘Bangladeshi’, ‘Indian’ and ‘Pakistani’) while others encompass a range of diverse, ethnic self-ascriptions (e.g. ‘White Other’ or ‘Black African’). While we recognise that ethnic categorisations are inherently subjective and contestable, we use the term ethnic in this paper for simplicity to refer to the 11 aggregate Census categories.

At this broad level of ethnic categorisation, the comparison between OM and Census yields information on how accurately a names-based ethnicity estimation predicts groupings of self-assigned ethnicity. Furthermore, we test whether prediction accuracy differs for different ethnic groups or other socio-demographic characteristics.

### 3.2. Screening: Names as markers of ethnic identity

We first explore the extent to which names act as markers of ethnic identity in England and Wales in the 2011 Census. In order to do so, the ONS VML was used to generate the relative frequency of each fore and surname in each of the 11 ethnic groups. We then fed these frequencies into a two-step clustering procedure [19], in which we used Ward’s hierarchical clustering to identify a parsimonious number of clusters from the resulting dendrogram. In so doing, we were guided by our objectives of developing reliable name-centric diagnostics, whilst also minimising the automated suppression of counts of ten or fewer occurrences by the VML facility. The cluster centres so identified were fed into *k means* cluster analysis. The dominance of a single ethnic group within a cluster was taken as a measure of how successfully a group of names predicts ethnic identity.

### 3.3. Evaluation: Diagnostics of prediction accuracy

We defined diagnostic tables to measure the estimation accuracy of OM (Table 1). We calculated two types of diagnostics: (1) name-wise diagnostics, which measure—for each name and ethnic group—the proportion of people whose ethnic group has been accurately predicted relative to all people bearing the same name; and (2) aggregate diagnostics, which summarise—for each ethnic group—prediction accuracy across selected demographic, social and geographic categories: age, gender, marital status and Government Office Region. Each result was provided subject to a minimum count threshold of 10 for reasons of disclosure control.

**Table 1:** List of tables provided as input into the VML, subject to minimum stated counts.

VML input table	value	min. count
**Name-wise diagnostics**		
1. forename by ethnicity	for each forename: n(correct) / n(bearers)	10
2. forename by age and ethnicity	for each forename: n(correct) / n(bearers in age group)	10
3. surname by ethnicity	for each surname: n(correct) / n(bearers)	10
4. surname by region and ethnicity	for each surname: n(correct) / n(bearers in region)	10
5. full name by ethnicity	for each full name: n(correct) / n(bearers)	100
**Aggregate diagnostics**		
6. sex by age and ethnicity	n(correct, sex, age, ethnicity) / n(sex, age, ethnicity)	10
7. sex by marital status and ethnicity	n(correct, sex, marital status, ethnicity / n(sex, marital status, ethnicity)	10
8. sex by region (GOR) and ethnicity	n(correct, sex, GOR, ethnicity) / n(sex, GOR, ethnicity)	10

## 4. Screening: Names and self-assigned ethnicity

Clustering fore and surnames reveals that both types of names are informative markers of ethnic identity. We define well-specified clusters as those that are dominated by a set of names of which at least 60 per cent of their bearers belong to only one ethnic group. These can be identified visually in cluster boxplots (provided in the supplementary material).

### 4.1. Cluster analysis of forenames

**Fig 1:**
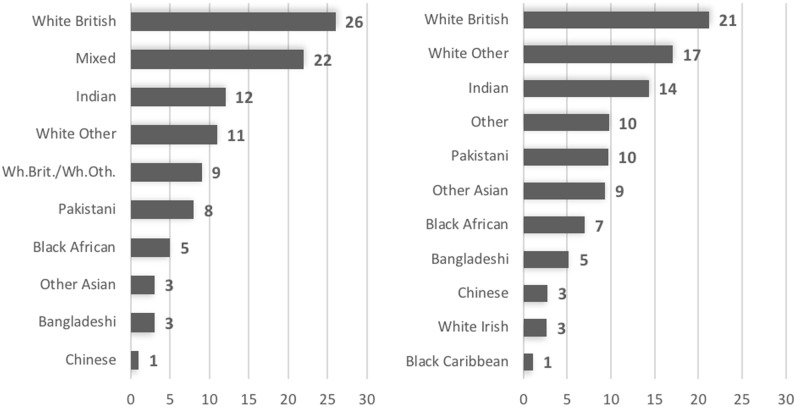
Proportion of forenames in different ethnic groups estimated from cluster analysis of names in the 2011 Census (left) and from predictions by Onomap (right).

The cluster analysis identifies 10 clusters of forenames, out of which eight are well defined (Fig 1 and S1 Fig). The most notable cluster comprises 26 per cent of forenames, which are mainly borne by individuals defining themselves as White British. Another 12 per cent is composed of names whose bearers overwhelmingly identify themselves as Indian, and 8 per cent appear to be Pakistani names. A share of 11 per cent identifies people that describe themselves as White Other.

Alongside these larger clusters, there are a number of smaller clusters that identify Black African (5 per cent), Bangladeshi (3 per cent) and Chinese (1 per cent) names to a high degree of certainty. On the other hand, 22 per cent of forenames cannot be attributed to any ethnic groups, although this cluster also includes a small number of names that emerge as White Irish. Another cluster of unclear ethnic attributions comprises names whose bearers variously describe themselves as White British or White Other. There are no forenames that can be attributed to Black Caribbeans.

Onomap, which assigns only one ethnic group to a name, generates a broadly similar distribution with some variation in the proportions of forenames. Onomap classifies 21 per cent of forenames as White British, compared to 26 per cent in self-assignment, and 17 per cent as White Other, compared to 11 per cent. Combining these classes, Onomap predicts 41 per cent of names to be White British, Irish or Other, while based on self-assignment, these total 47 per cent including the mixed category White British/White Other. Onomap marginally over-estimates the number of Bangladeshi, Chinese, Indian, Pakistani and Black African names (each by 2 per cent) compared to self-assignment. The category Other Asian is significantly larger, while the catch all Other category is just 10 per cent. In total, Onomap predicts 41 per cent of forenames to be Asian, compared to only 27 per cent from self-assignment. It is probable that the Mixed category contains names that Onomap classifies as Asian or Black.

### 4.2. Cluster analysis of surnames

**Fig 2:**
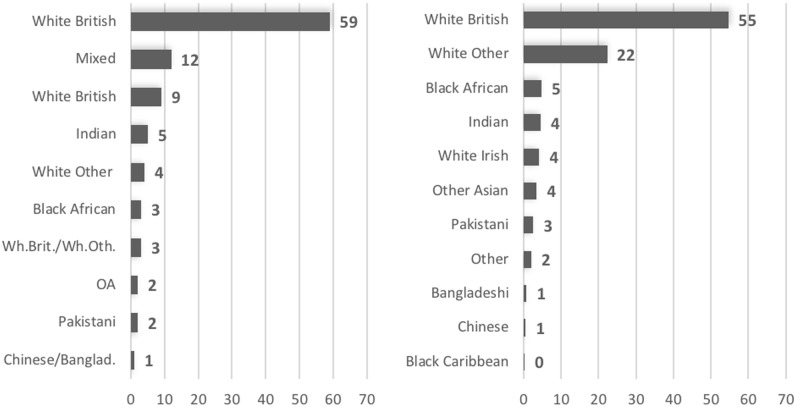
Proportion of surnames in different ethnic groups estimated from cluster analysis of names in the 2011 Census (left) and from predictions by Onomap (right).

The cluster analysis applied to surnames identified ten clusters, too. Seven out of these ten are well-specified in terms of ethnicity (Fig 2 and S2 Fig). 59 per cent of surnames are assigned to the White British category with a probability of over 90 per cent. Another cluster with 9 per cent of surnames can also be attributed to White British, albeit with lower probabilities of between 65 and 80 per cent. This is still a well-specified cluster according to our criteria. The remaining well-specified clusters comprise names of people who describe themselves as Indian (5 per cent), White Other (4 per cent), Black African (3 per cent), Pakistani (2 per cent) and Other Asian (2 per cent). A share of 12 per cent of surnames cannot be attributed to a single ethnic group, and two smaller clusters comprise names which are shared by two ethnic groups: White British and White Other (3 per cent) and Chinese and Bangladeshi names (1 per cent). The latter cluster, however, shows higher probabilities in identifying these ethnic groups, and hence, division of this cluster may separate Bangladeshi and Chinese names. However, given the limit to the queries that could be processed through the VML link, this step has not been undertaken.

Similar to the cluster analysis of self-assignment, Onomap classifies 55 per cent of surnames as White British but significantly more surnames as White Other compared to the self-assignment cluster analysis– 22 versus 4 per cent. Onomap assigns 81 per cent of all surnames to one of the White categories, with a corresponding figure of 75 per cent in the cluster analysis of self-assignments. The proportions of Asian surnames predicted by Onomap range from 0.5 (Chinese) to 4.5 (Indian) per cent, figures which are consistent with the cluster analysis of self-assignments. Onomap assignments to the Other category are significantly smaller in number than in the Mixed category of the cluster analysis, which indicates that some of Onomap inaccurately infers an ethnic group for names with diverse subjective affiliations.

### 4.3. Using names to estimate ethnicity

The results suggest that names widely correlate with subjective, ethnic identity. Nevertheless, forenames and surnames relate to ethnicity differently. Whereas nearly 70 per cent of surnames clearly relate to White British self-identification, only 26 per cent of forenames do so. Nearly one third of forenames cannot be attributed clearly to one ethnic group; for surnames, this share drops to 14 per cent. Therefore, surnames offer better prospects to be used as markers of ethnic self-identity than forenames.

Yet some ethnic groups appear to be poorly identified by the mix of names in England and Wales. In all cases, the Black Caribbean, White Irish and Other groups are scattered between clusters. Potentially, these groups can be better identified when both forenames and surnames are taken into account. Other groups of names that span multiple ethnic groups are likely a result of stronger cultural links and cultural integration over time, notably between White British and Other White. A joint view of classes assigned to forenames and surnames may better distinguish individuals from these groups.

## 5. Evaluation and analysis of prediction accuracy

Aggregate prediction accuracy across age group, marital status and Government Office Region deliver clues if naming practices vary according to social or geographical context. If variations can be found, these need to be considered in the advancement of names-based classifications that better consider elements of subjective identity. Table 2 provides the absolute population counts of different, self-assigned ethnic categories in the Census. The population counts offer a robust basis to compare prediction accuracy; furthermore, they represent the entire population on Census night (where name was captured with sufficient accuracy and ethnicity was not imputed).

**Table 2:** Frequencies of aggregate categories of self-assigned ethnicity in 2011 Census (England and Wales) rounded to the nearest 100 from calculations made using VML.

**White British**	**White Irish**	**Other White**	**Indian**	**Pakistani**	**Bangladeshi**
41,764,000	484,300	2,242,200	1,328,400	1,047,900	403,800
**Chinese**	**Other Asian**	**Black African**	**Black Caribbean**	**Other**	**Missing (in Census)**
349,100	686,100	884,900	532,000	1,366,800	688,700

### 5.1. Socio-demographics: Gender, age and marital status

**Fig 3:**
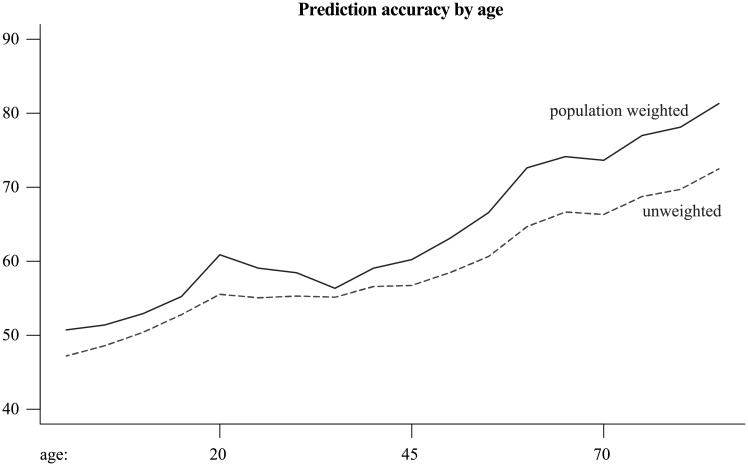
Unweighted prediction accuracy for different age bands.

Viewed across all classified persons in the Census, Onomap better predicts the ethnic affiliation of older individuals (Fig 3). Across all forenames, the average prediction accuracy improves from 47 to 70 percent between the lowest and the highest age band. If names are weighted by the number of their bearers, these rates improve to 50 and 80 percent at the lowest and the highest age band respectively. This is potentially concerning; it implies that half of all predictions for participants below age 20 are incorrect. This pattern suggested that, if self-assignment data are not available, age should be considered in algorithmic approaches when devising an improved classification.

**Fig 4:**
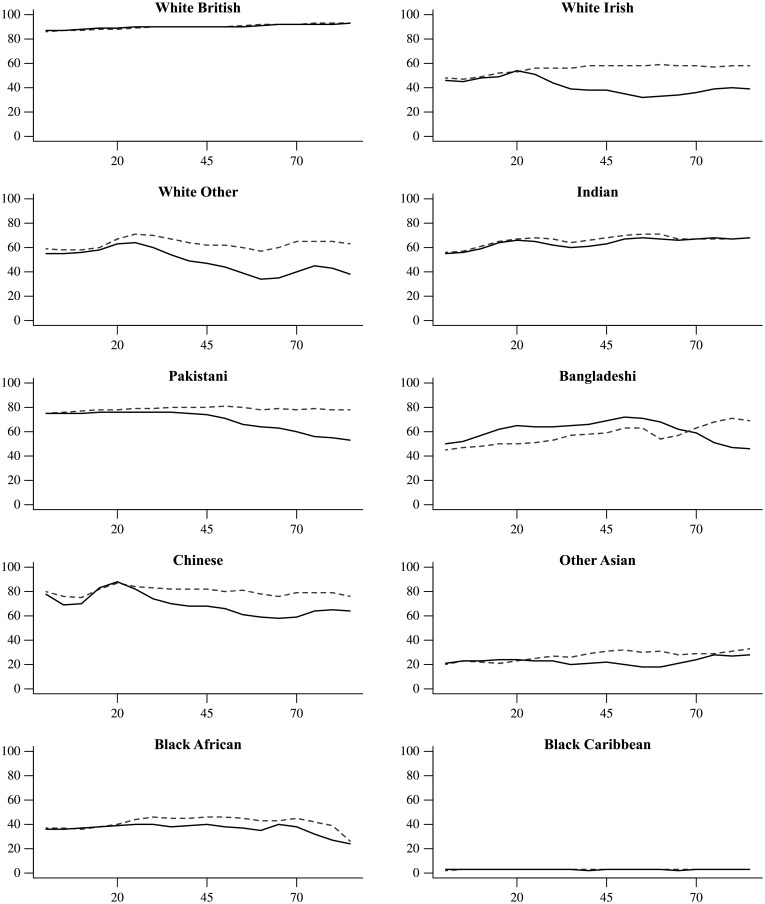
Prediction accuracy for women (solid line) and men (dashed line) by age in each self-ascribed ethnic category (the small Arab/Mixed/Other category is not shown).

The over-all age-related pattern of prediction accuracy masks significant variations by gender and ethnic group (Fig 4). The highest rates can be observed for White British and Chinese, the lowest for Black Caribbean, Black African and Other Asian. For many ethnic groups, prediction accuracy improves with age. The range of accuracy rates across age groups spans the 20 to 40 percent range. Yet for White Irish, White Other, Pakistani and Black African younger names are better predicted. Onomap performs consistently better for men than for women. The gender gap only becomes apparent beyond age 20 in most ethnic groups, however. This is likely to mark the point of marriage and spousal surname adoption. Bangladeshi names are an exception to this pattern; here, Onomap predicts more accurately for women up to age 70.

**Fig 5:**
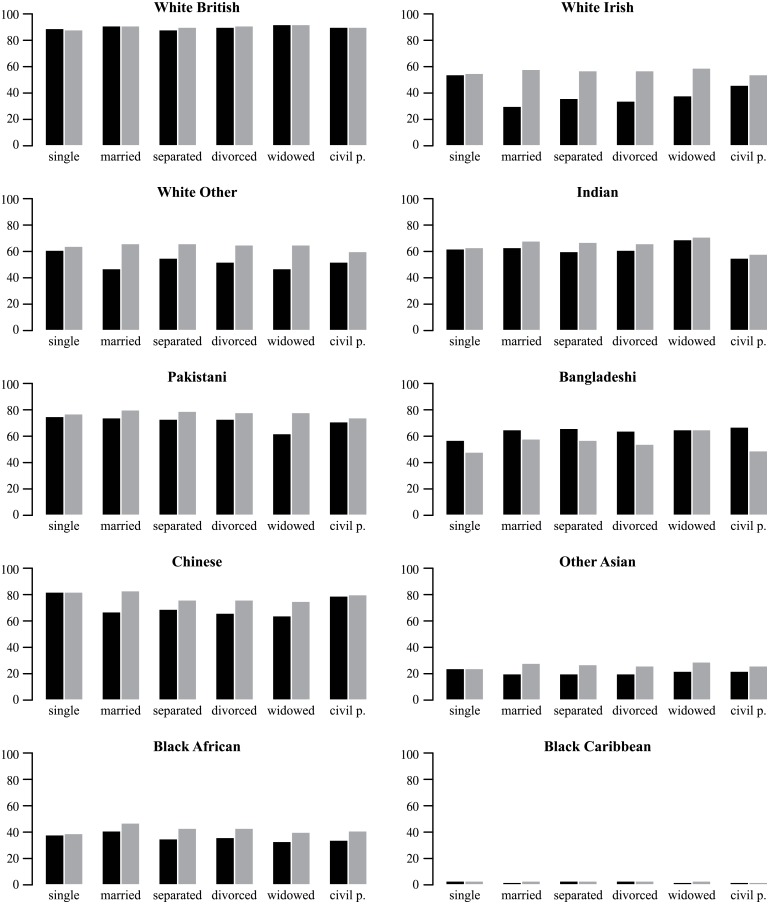
Prediction accuracy for women (black bar) and men (grey bar) by marital status in each self-ascribed ethnic category (the small Arab/Mixed/Other category is not shown).

Prediction accuracy varies by marital status in accordance with age (Fig 5). There is little difference in prediction accuracy between men and women among single persons, except for Bangladeshi individuals, where female names were better predicted than male names. Prediction accuracy is lower for married women than for married men. This pattern is strongest for White Irish, White Other and Chinese. There is no difference in prediction accuracy for married White British individuals. The reason for this may be the frequency of marriage between individuals that are both White British, dominating other marriages across ethnic groups. The lower prediction accuracy found for married women persisted for separated, divorced and widowed women in those groups where marriage exhibited gender differences.

### 5.2. Geographic variation by Government Office regions

**Fig 6:**
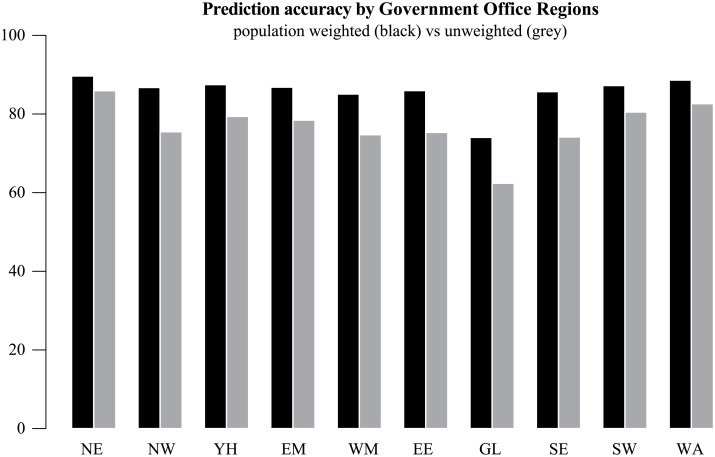
Prediction accuracy according to region of residence (North East NE; North West NW; East Midlands EM; West Midlands WM; Yorkshire and Humber YH; East England EE; Greater London GL; South East SE; South West SW; Wales WA).

Prediction accuracy rates using Onomap across Government Office regions vary between 60 and 85 per cent of names (Fig 6). If the population rather than names are taken into account, in all regions except Greater London, the tool predicts 90 per cent of individuals’ ethnic groups correctly.

**Fig 7:**
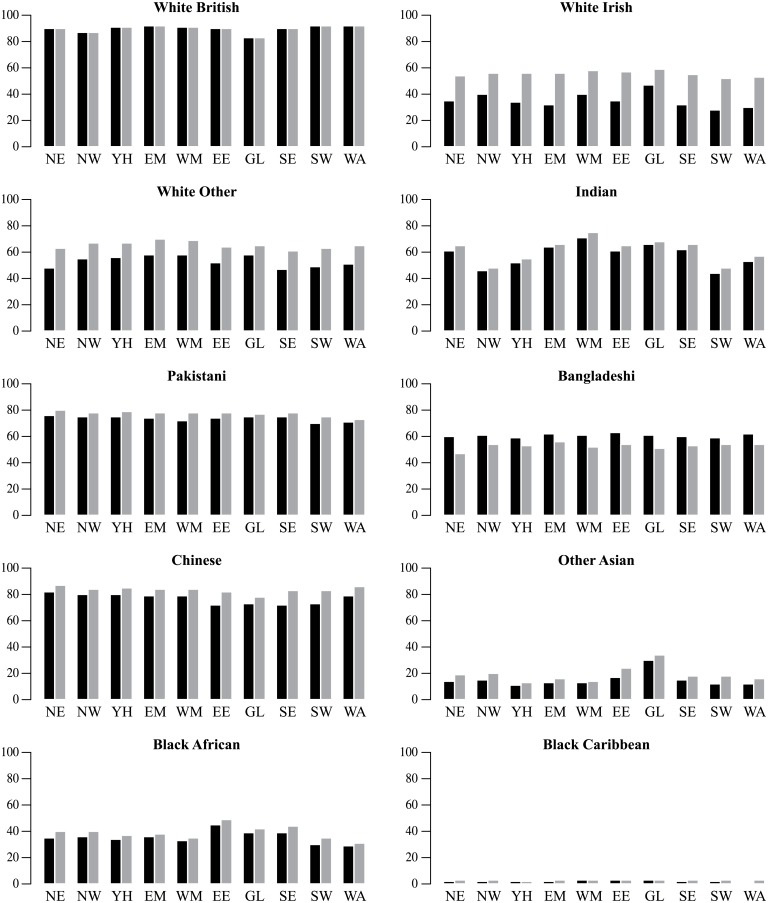
Prediction accuracy for women (black bar) and men (grey bar) by region in each ethnic group (codes for regions as in Fig 6).

The over-all pattern of higher prediction accuracy outside Greater London reflects underlying ethnic geographies (Fig 7). White British are consistently well predicted (above 90 percent), except in Greater London (GL), where prediction accuracy is closer to 80 percent. Chinese are also less often correctly predicted in Greater London. By contrast, occurrences of Greater London’s Irish, other Asian and Black African populations are more successfully predicted. Greater prediction accuracy for Black Africans can also be found in East England (EE) and the South East (SE). There were some other regional patterns with prediction of Indian ethnicity most successful in the West Midlands (WM: 71% women, 75% men) and least successful in the South West (SW: 44% women, 48% men) and the North West (NW: 46% women, 48% men). Regional variation in prediction accuracy of White Irish women is more pronounced than for men.

### 5.3. Implications for ethnicity estimation from names

We have undertaken evaluation of Onomap as an exemplar of unsupervised, machine-learning classifications in the expectation that similar methods will share vulnerabilities with respect to self-assignments. Use of 2011 Census data shows that the accuracy of names-based ethnic classification in predicting self-assigned ethnicity varies with a number of socio-demographic and geographical characteristics of individuals. Onomap is more accurate for older individuals, although there are important variations among ethnic groups. For individuals below the age of 20, the accuracy can drop to 50 per cent. As naming practices become more cosmopolitan, the prediction accuracy of Onomap is likely to reduce further for younger ages in the future. Prediction accuracy is higher for men than for women after age 20. The gap between men and women varies by ethnic group; this trend is most pronounced for the White Irish.

In terms of geography, we find that Onomap successfully predicts some ethnic groups less often and others more often in highly diverse, urban regions, notably Greater London. This may be because names provide better indications of ethnicity for migrants recently arrived in cities, while in other regions, longer settled migrant populations may come to adopt non-British names. Across all regions, Onomap usually fails to identify Other Asian and Black groups.

Taken together, these findings illustrate the uncertainty inherent in data driven classifications using forename-surname pairings alone. It also suggests the following priorities for classification where data on name bearers’ self-assigned ethnicity are available.

1. **Probabilistic assignment.** Data driven cluster solutions such as Onomap are most clearly interpreted deterministically, yet probabilistic assignments provide a more satisfactory interpretation of the inherent uncertainties, particularly in the context of subjective self-assignment.
2. **Separate classifications for fore and surnames**. Users should be provided with the option of using prediction based purely upon forenames, or surnames, or some combination of the two. This would be useful if, for example, data for classification were known to include many individuals of mixed ethnicity or are of second or subsequent migrant generations.
3. **User input of age, gender or marital status**. Our evaluation confirms that age, gender and marital status are likely to provide useful ancillary information to improve prediction accuracy of self-assigned ethnicity.

## 6. Ethnicity Estimator development and evaluation

Following the evaluation of Onomap, we seek to develop an advanced ethnicity estimator that improves the prediction of self-assigned ethnic identity as recorded in the 2011 Census of England and Wales. We define new, enhanced algorithms that incorporate the recommendations resulting from the evaluation. As with Onomap, we evaluate each of these algorithms by analysing aggregate diagnostics of prediction accuracy provided by ONS in the VML. Informed by these results, we specify a second, refined set of algorithms and evaluate them using the same procedure as above. As a final outcome, we select the algorithm that best predicts self-assigned ethnicity and build a freely available tool that is optimised for application in England and Wales. The tool is available for research for the public benefit upon application and review.

### 6.1. Definition of Onomap and Ethnicity Estimator algorithms

Based on the findings of the Onomap evaluation, we define a set of further ‘Ethnicity Estimator’ (EE) algorithms, which each incorporate an additional feature corresponding to the recommendations formulated earlier (Table 3).

**Table 3:** First set of follow-up algorithms and their main characteristics.

label	Description
**Follow-up algorithms using the Onomap lookup tables**	
OM-F	Ethnicity estimation for a person based on forename ethnicity as recorded in Onomap (= OM)
OM-G	… based on fore or surname ethnicity depending on gender of person (using Onomap)
OM-GA	… based on either fore or surname based on gender and age of record (using Onomap)
**Follow-up algorithms using new Census-based lookup tables (detailed explanation in text)**	
EE-A	… based on highest mean of the forename and surname ***weights*** of matching ethnicities (aka additive)
EE-M	… based on highest product of the forename and surname ***weights*** of matching ethnicities (aka multiplicative)
EE-R	… based on that combination of forename and surname matching ethnicities with the highest surname ***rank***.
EE-RS	… based on highest surname ***rank*** (see text)
EE-W	… based on highest ***weight*** (see text) for forename or surname

The OM algorithms use the original Onomap lookup tables. OM-F is different from the original Onomap (OM) as it gives precedence to the forename ethnicity. OM-G and OM-GA use additional information to estimate ethnicity when forename and surname assignments differ. OM-G uses the ethnic group of the forename when a person is female and the surname group when a person is male. This feature is to account for the lower reliability of female surnames described in Section 5 (Fig 7). OM-GA uses the ethnic group of the forename when a person is female and younger than 20, the group of the surname when a female person is 20 years or older, and surname group in all other cases. This is to account for the surname adoption of women upon marriage.

The new algorithms of ethnicity estimation (EE) use the relative frequencies of the 11 Census ethnic groups for each name. For example, if 81% of people with surname AARON describe themselves as ‘White British’, 6% ‘Black Caribbean’, 5% ‘Other’ and 4% each ‘White Other’ and ‘Black African’, a table block is defined for surname AARON with the relative frequencies for each ethnic group recorded as weight (Table 4). This procedure is repeated for each surname until a complete long-table of all surnames and their weights is created. An equivalent table is produced for forenames.

**Table 4:** The Census-based surname lookup table for Ethnicity Estimator.

name	rank	ethnicity code	ethnicity label	weight
AARON	1	WBR	White British	.81
AARON	2	BCA	Black Caribbean	.06
AARON	3	OXX	Other ethnic group	.05
AARON	4	WAO	White Other	.04
AARON	5	BAF	Black African	.04

Using this information, the new EE algorithms differ with respect to the way in which they assign an ethnic class based on these long-tables (see example in Table 5). EE-A calculates the mean weight of matching forename-surname ethnic classes and assigns the ethnic class with the highest mean weight to the person to be classified. EE-M multiplies the weights of matching forename-surname ethnic class and chooses the class with the highest value. EE-R selects that ethnic class which involves the highest rank of either fore and surname. If there is a tie for two classes, it selects the class involving the highest surname rank. EE-RS does not compare forename and surname ethnicity: rather, it always selects the ethnic class with surname rank is 1. EE-W assigns that ethnic class which shows the highest weight across fore and surname.

**Table 5:** Example of assigning ethnic class using Ethnicity Estimator.

forename	surname	matched class	forename		surname		product	mean	assignment by EE				
			weight	rank	weight	rank			-A	-M	-R	-RS	-W
SHERNETTE	AARON	WBR	.07	2	.81	1	.0567	.44	-	X	X	X	
SHERNETTE	AARON	BCA	.84	1	.06	2	.0504	.45	X	-	-	-	X
SHERNETTE	AARON	OXX	-	-	.05	3	-	-	-	-	-	-	-
SHERNETTE	AARON	WAO	-	-	.04	4	-	-	-	-	-	-	-
SHERNETTE	AARON	BAF	-	-	.04	5	-	-	-	-	-	-	-

### 6.2. Evaluation of algorithms using Census data

We compare aggregate prediction accuracy of all new algorithms against the original Onomap algorithm (OM) as a benchmark. A share of 89.9 per cent of all individuals defining themselves as White British (WBR) are accurately predicted. All EE algorithms accurately predict ‘White British’ to at least 99.0% (Table 6).

**Table 6:** Aggregate prediction accuracy of all algorithms for each ethnic group.

algo.	ethnic group											weighted*		unweighted	
	WBR	WIR	WAO	AIN	APK	ABD	ACN	AAO	BAF	BCA	OXX	WBR-OXX	WAO-OXX	WBR-OXX	WAO-OXX
OM	89.88	**47.34**	**59.11**	64.24	76.13	56.45	78.51	23.61	39.71	2.90	5.87	82.57	46.83	**49.43**	45.17
OM-F	84.47	42.14	55.09	61.13	70.81	44.13	59.97	22.56	32.20	4.13	**8.93**	77.42	43.06	44.14	39.88
OM-G	88.61	47.16	57.80	62.68	72.09	49.43	69.24	23.37	35.88	3.33	7.30	81.20	44.89	46.99	42.35
OM-GA	89.81	47.75	58.73	64.20	75.97	54.20	73.00	23.98	37.42	3.31	6.24	82.42	46.26	48.60	44.12
EE-A	99.54	0.01	43.21	76.58	87.63	59.61	66.22	32.30	45.42	3.63	3.03	**90.09**	46.54	47.02	46.40
EE-M	99.54	0.01	43.14	76.39	**87.97**	58.93	66.15	31.97	45.22	3.43	3.46	90.08	46.51	46.93	46.30
EE-R	99.28	0.07	43.89	**76.61**	86.27	**60.11**	68.28	33.12	**48.27**	4.90	4.09	89.98	**47.22**	47.72	47.28
EE-RS	99.01	0.02	43.53	75.59	85.11	56.77	**81.55**	**34.02**	48.19	**5.13**	2.20	89.72	47.01	48.28	**48.01**
EE-W	**99.63**	0.01	42.94	75.96	86.65	59.96	70.90	32.05	44.07	2.40	1.70	90.08	46.05	46.93	46.29
**%**	83.00	0.95	4.05	2.46	2.03	0.77	0.68	1.12	1.48	1.02	2.44				

* means of average prediction accuracy across all ethnic groups, weighted by relative population size (last row); the unweighted figures provide average prediction accuracy regardless of ethnic group size.

By contrast, the EE algorithms hardly predict any Irish individuals accurately whereas Onomap achieves rates of nearly half. Indeed, Onomap-based algorithms predict ‘White’ and ‘Other’ ethnic groups better, whereas EE better predict ‘Asian’ and ‘Black’ ethnic groups. Improvements over Onomap are substantial for individuals describing themselves as ‘Indian’, ‘Pakistani’, ‘Other Asian’ and ‘Black African’.

On the whole, the additive EE algorithm (EE-A) performs best when all ethnic groups are taken into account and weighted by their population in the Census. In this case, average prediction accuracy is 90.1% for EE-A and 82.6% for OM. When ethnic groups are not weighted, OM fares best. When ‘White British’ and ‘White Irish’ are excluded from these metrics, the rank-based EE-R and EE-RS fare best depending on whether the metric is weighted or unweighted.

The main findings from this evaluation are:

- Contrary to our expectations (see section 5), adding age to estimate ethnicity does not improve prediction accuracy. It is thus likely that Onomap’s relative lack of success in predicting ethnicity of younger individuals arises because its name dictionary is confined to those who were at least 17 years old in 2007.
- The EEs predict ‘White British’ extremely well. Since this is by far the most frequent ethnic group, EEs are far superior in the weighted summary metrics.
- The OM algorithms are superior in detecting non-British White groups.
- Among the EEs, the weights-based algorithms (EE-A, EE-M and EE-W) produce very similar results. Among these, the additive EE (EE-A) fares best on both weighted and unweighted metrics.

The entire evaluation procedure repeated for each algorithm produces a wide range of results, and we will only highlight a few main points here.

Using the forename—regardless of whether it is used alongside gender or age—improves prediction accuracy at older ages (except for ‘White British’) and reduces prediction accuracy at younger ages in OM-F and OM-G. This pattern applies particularly for men, although greater emphasis on forenames significantly worsens prediction accuracy for Chinese and Black Caribbean.

**Fig 8:**
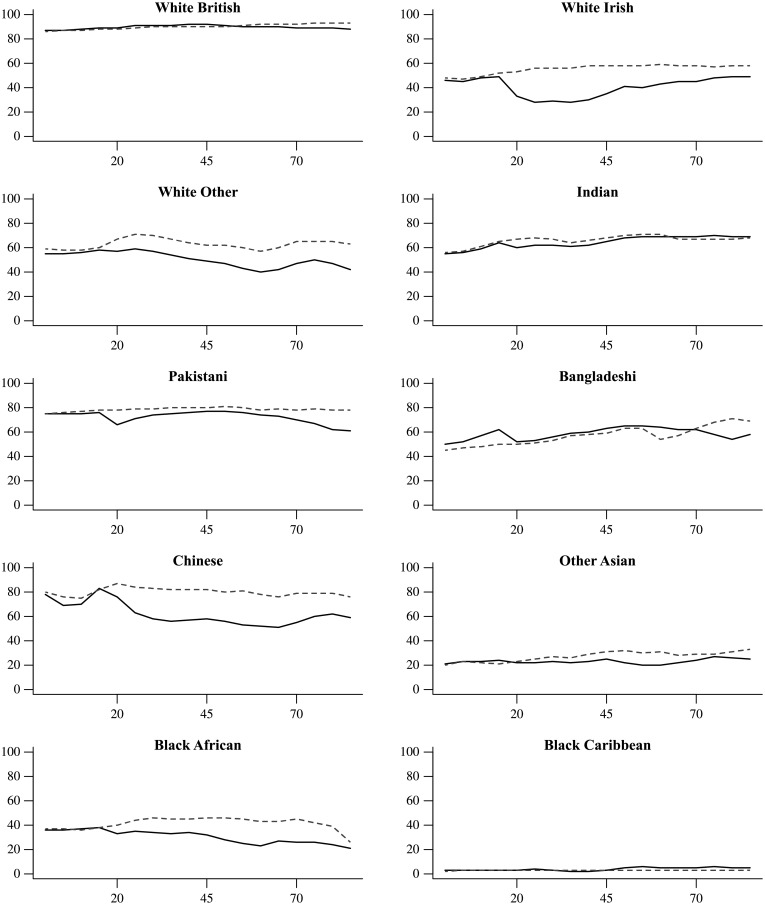
Prediction accuracy of OM-GA for women (solid line) and men (dashed line) by age in each ethnic group.

Adding age as a criterion of assigning ethnicity (OM-GA) does not lead to noticeable improvements of prediction accuracy over and above the inclusion of gender. Indeed, the predictions accuracy for White Irish women older than 20 drops to half of its unadjusted prediction accuracy and similar though less pronounced patterns can be found for Chinese, Black African and White Other (Fig 8). Some exceptions are married or divorced Pakistani and Bangladeshi women, for whom prediction accuracy mildly increases upon inclusion of age and gender. Neither do age and gender affect regional patterns of prediction accuracy.

The pattern of declining prediction accuracy with increasing age persists for all EE algorithms. Regional patterns of prediction accuracy persist in the EE algorithms, too, although they are attenuated for most ethnic groups compared to OM. For Indian, White Other and Chinese, however, accuracy is lower in Greater London and SE England than in OM. There is no discernible improvement in accuracy for marital status.

In summary, addition of demographic or geographic criteria in predicting self-assigned ethnicity add little improvement, and the additional computational complexity they introduce does not seem to be justified. In fact, an age factor reduces prediction accuracy for women of older age cohorts. We can further conclude that surnames are superior in predicting ethnicity than forenames for most ethnic groups, especially for Chinese, but they are helpful in discerning related ethnic groups, such as White British and White Other. Whilst it is unsurprising that surnames are the best components for estimating likelihood of membership of a particular ethnic group, there is also scope for weighting their importance when seeking to maximise over-all prediction accuracy: we address this issue below.

### 6.3. Name format

Census data on names are often incomplete or not captured accurately using Optical Character Recognition (OCR) procedures. Accordingly, we examine the pattern of prediction accuracy where individual names are incomplete, and consider the degree of concordance between forename and surname markers for the different ethnic groups (Table 7).

**Table 7:** Instances of incompleteness of forename and surname pairs, and types of inconsistency between forenames and surnames.

code	Description
_s	forename missing, surname available
f_	forename available, surname missing
fs	both forename and surname available
1u	fore and surnames have no possible ethnic group in common
con	consonant—both forename and surname are classified as same census ethnicity
dis	dissonant—forename and surname are classified as different
com	surnames are composites, either hyphenated or spaced (e.g. ‘Brown-Taylor’, ‘De Smith’)
mis	surname missing
sgl	surnames only have single component (e.g. ‘Brown’, ‘Smith’, ‘Taylor’, ‘O’Brien’)
su	surname not matched

**Fig 9:**
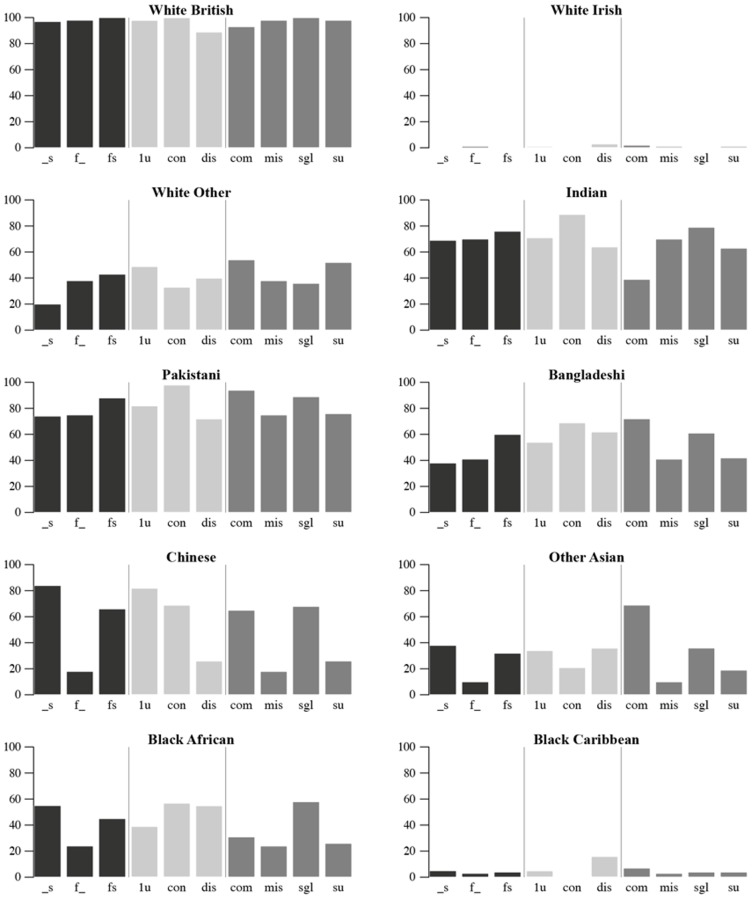
Prediction accuracy of EE-A by format of names in each ethnic group.

While prediction accuracy remains high for White British, the rate drops to 85 per cent when names are dissonant (Fig 9). For White Other, prediction accuracy appears to be better when surnames are missing or when fore and surnames are dissonant. For Bangladeshi, Indian and Pakistani, forenames and surnames seem to be equally consistent markers for ethnicity. For each of these ethnic groups, prediction accuracy drops when fore and surname classes are dissonant. Composite surnames offer better prediction among Pakistani and Bangladeshi individuals, indicating that composites are more common in these ethnic groups. Surnames are by far more successful markers for Chinese, Other Asian and Black African. Accordingly, prediction accuracy drops strongly for these groups when the surname is missing.

In summary, prediction accuracy associated with the format of names reflects the different roles of fore and surnames in the naming practices of different ethnic groups. A potential modification informed by these results might be to adjust weights between fore and surnames depending on ethnic class and format. For example, surname weights could be adjusted upward where a name can be Chinese, Other Asian or Black African or where a name can be Pakistani and is composite. An optimisation algorithm might usefully identify optimal weights for this procedure. Given the complexity of format-based weight adjustments in the VML context, this idea has not been further pursued at this stage.

### 6.4. Final refinement and selection of Ethnicity Estimator

In view of the findings from this evaluation, the objective for final refinement of Ethnicity Estimator is to increase the detection sensitivity to ethnic groups other than White British, in particular to improve prediction accuracy for White Irish, White Other and Black Caribbean. Therefore, we adjust the weights for White British downward to different degrees. The impact of the downward adjustment is not only that non-‘White British’ groups gain in importance relative to White British but also that White British receives a lower rank in most table blocks pertaining to a name. Thus, all EE algorithms, which either use weights or ranks, will be affected by the weights adjustment. Because EE-A and EE-RS emerged as the best predictive algorithms among EEs, and EE-R is not sensitive to weights adjustment beyond what is captured in EE-RS, we evaluate a new set of weights for EE-A and EE-RS (Table 8).

**Table 8:** Re-weighted modifications of algorithms to mitigate the dominance of the ‘White British’ category for EE-A and EE-RS.

label	description	surname weight	forename weight
**additive (EE-A)**			
EE-A1	EE-A with WBR re-weighted (surnames)	1/3	1
EE-A2	EE-A with WBR re-weighted (surnames)	1/9	1
EE-A3	EE-A with WBR re-weighted (fore and surnames)	1/3	1/3
EE-A4	EE-A with WBR re-weighted (fore and surnames)	1/9	1/3
EE-A5	EE-A with WBR re-weighted (fore and surnames)	1/3	1/9
EE-A6	EE-A with WBR re-weighted (fore and surnames)	1/9	1/9
**highest surname rank (EE-RS)**			
EE-RS1	EE-RS with WBR re-weighted (surnames)	1/3	1
EE-RS2	EE-RS with WBR re-weighted (surnames)	1/9	1

The summary metrics suggest that the re-weighted EE with a 9-fold downward adjustment of White British weights for both forenames and surnames (EE-A6) performs best (Table 9). We can observe the most important improvements in prediction accuracy rates for White Irish from nearly 0 to 22 per cent, White Other from 43 to 65 per cent, now outperforming OM, Chinese from 66 to 78 per cent, Black Caribbean from 4 to 16 per cent and Other from 3 to 11 per cent, now outperforming OM in this respect, too. As expected, prediction accuracy rates for White British drop but still stand at 95 per cent.

**Table 9:** Aggregate prediction accuracy of re-weighted algorithms for each ethnic group.

algo.	ethnic group											weighted*		unweighted	
	WBR	WIR	WAO	AIN	APK	ABD	ACN	AAO	BAF	BCA	OXX	WBR-OXX	WAO-OXX	WBR-OXX	WAO-OXX
**OM**	89.88	47.34	59.11	64.24	76.13	56.45	78.51	23.61	39.71	2.90	5.87	82.57	46.83	49.43	45.17
**EE-A**	99.54	0.01	43.21	76.58	87.63	59.61	66.22	32.30	45.42	3.63	3.03	90.09	46.54	47.02	46.40
**EE-A1**	**99.00**	1.00	46.00	78.00	89.00	60.00	68.00	33.00	46.00	5.00	4.00	89.89	48.07	57.18	47.67
**EE-A2**	**99.00**	4.00	47.00	79.00	89.00	**61.00**	70.00	34.00	46.00	8.00	4.00	90.05	48.87	58.27	48.67
**EE-A3**	**99.00**	2.00	55.00	79.00	89.00	**61.00**	71.00	34.00	50.00	8.00	4.00	**90.42**	51.29	59.27	50.11
**EE-A4**	98.00	7.00	56.00	80.00	**90.00**	**61.00**	78.00	**35.00**	51.00	11.00	5.00	89.85	52.63	61.09	51.89
**EE-A5**	97.00	7.00	64.00	80.00	**90.00**	**61.00**	72.00	**35.00**	**53.00**	11.00	9.00	89.43	55.18	61.73	52.78
**EE-A6**	95.00	**22.00**	**65.00**	**81.00**	**90.00**	**61.00**	78.00	**35.00**	**53.00**	**16.00**	**11.00**	88.12	**56.47**	**64.27**	**54.44**
**EE-RS1**	98.00	1.00	49.00	77.00	86.00	57.00	83.00	**35.00**	49.00	8.00	2.00	89.23	49.09	58.64	49.56
**EE-RS2**	95.00	**22.00**	51.00	79.00	86.00	58.00	**86.00**	**35.00**	50.00	**16.00**	3.00	87.22	50.83	61.91	51.56
**%**	83.00	0.95	4.05	2.46	2.03	0.77	0.68	1.12	1.48	1.02	2.44				

* weighted average across the ethnic groups shown weighted by the relative frequency of each ethnic group shown in the bottom row. High values column-wise values are highlighted in bold. Note that EE values for individual ethnic groups were rounded by ONS.

The final Ethnicity Estimator product employs an additive procedure to decide the ethnicity of an individual based on forename and surname, where available, with a 9-fold downward re-weighting for White British. Given a forename-surname pair of an individual, the algorithm calculates the mean of the weights for forename and surname for each matching ethnic class. The ethnic class that yields the highest mean weight is then selected as the predicted class.

## 7. Conclusions

### 7.1. Predicting self-assigned ethnicity from names

The screening and evaluation demonstrates that, in a significant number of cases, there is not a single answer to which ethnic group a name properly belongs. Ethnic profiles of names are likely to become more diverse as cultural integration continues and naming practices become more cosmopolitan. We are likely to observe the effect of cosmopolitan naming practices in urban areas, such as London, where prediction accuracy is lower. On the other hand, cities often act as gateways for newly arrived immigrants, and therefore, ethnic identities might be more pronounced within these groups. The observed higher level of self-identification as Irish in London may support this hypothesis.

In most cultures, surnames are adopted, retained or abandoned according to defined social norms, whereas forenames are given under fewer social constraints. As a consequence, surnames make better markers of ethnicity, whereas forenames provide useful, additional information to distinguish more related ethnic groups, such as White British and White Other. Nevertheless, the relationship between ethnic identity and naming practices are inherently time and cohort-specific as they reflect processes of integration, assimilation and taste [14]. Names classifications are necessarily historical and need to be continuously updated. The ease of updating lookup tables based on highly granular, secure data places an important advantage of the Ethnicity Estimator algorithms over machine-learning solutions, whose entire structure would need to be re-built each time as they depend on pattern detection in the full population.

Cohort-specific effects may be most clearly manifest with respect to individuals assigned by OM to the ‘White Irish’ category, who have surnames originating in Ireland and whose parents may have retained a past generation’s preference for Irish forenames. These individuals may nevertheless today self-identify as White British. It may also be that many Census respondents do not identify with nationalities subsumed within the ‘White Other’ category for the same reason, particularly where they are physically indistinguishable from the majority ‘White British’ category.

On the other hand, the size of pan-ethnic categories, such as White Other or Other Asian, limits their informational value. These categories were defined by the Census largely for statistical purposes. Perceived ethnic identities focus upon finer distinctions, such as nationalities or even sub-parts thereof. Indeed, it should be remembered that ethnic identity only forms one element of complex, nuanced and fluid social and cultural identities that people adopt in everyday life. In this paper, we view identity through the lens of the 2011 Census questionnaire, which nevertheless provides a framework within which to consider other forms and moments of social identification.

Our final classification procedure incorporates major recommendations resulting from the evaluations, specifically the separate classification of fore and surnames and probabilistic assignment based on empirical weights. Based on this work, we have also developed a free software product that classifies lists of names into the 11 Census ethnic groups, subject to small number disclosure controls required following ONS ethical review of this research. This is available to approved registered users at ee.cdrc.ac.uk.

The foregoing derivation and empirical analysis demonstrates that the Ethnicity Estimator offers an improvement to the Onomap classification and may offer similar advantages over other purely algorithmic approaches. The EE also provides a framework within which such approaches might be used to further partition the 11 principal Census categories. The research is unique in its use of cutting edge secure data access methods to devise and validate a predictively successful tool. For the first time, a names-based classification has been applied to an entire population and adjusted to account for subjective ethnic identity. The existing EE product clearly demonstrates that the imaginative re-use of government statistical data offers considerable advantages over purely data driven approaches to names classification.

We are undertaking a number of extensions to this research. First, in recognition that forenaming conventions are dynamic, we are extending and updating our own directories of names across the world, in recognition that at least part of Onomap’s limitations arise from its dependence upon data collected for adults only in or prior to 2007 [20]. Second, we are using the EE as a platform for disaggregation of non-Census categories. Third, we are refining the White British category into sub-classes of surnames reflecting their long-term geographic origin within the United Kingdom.

### 7.2. Limitations and outlook

Some extensions to the preceding analysis were undertaken but were only partial in coverage because of restrictions upon the analysis that could be undertaken in the VML. In particular, we identified age profiles associated with different given names. Unfortunately, the resources available did not permit a full evaluation—but in the future, it will be possible for the tool to make conditional predictions of gender and age alongside ethnicity from forenames. As for the latter, a focus upon forenaming practices among different ethnic groups may provide insights into trends of cultural assimilation [14].

As indicated at various points in this paper, the novel approach of secure and collaborative processing in a safe haven environment can be very time-consuming and labour-intensive and, as a consequence, many avenues for model refinement and optimization remain unexplored. A valuable extension would include the implementation of ethnicity specific optimization procedures to refine the weights, and a more comprehensive analysis of the correspondences between forenames and age and gender for different ethnic groups. In addition, implementation of fuzzy matching techniques, as suggested by [17], may improve the estimate of the ethnic profile associated with a name. While the pioneering use of ONS’s secure environment allowed indirect access to personal data, there was a limit—at this stage—on the number and nature of queries that could be undertaken. It may be possible in future to implement algorithms for fuzzy matching, optimisation of weighting schemes and more robust quality measures, such as precision rather than accuracy.

Finally, we are aware that the patterns of self-assigned ethnicity are contingent to England and Wales in 2011, and in important respects are likely to reflect not only the way that ethnicity was recorded in the 2011 Census but also the patterns and processes that characterize those countries at that time. It is important to use these results as an updateable platform for classification of other areas of the world.

In summary, we have demonstrated that names can be used to estimate subjective, ethnic identity. As far as we are aware, there is no other tool which is specifically designed for this purpose. We believe that identity-focussed classifications can offer important insights that are of use in policy domains that are concerned with social integration and ethnic inequalities, especially when these are repeatedly applied over time. Changes in prediction accuracy can further deliver evidence of differential processes of integration and assimilation and highlight the fluid boundaries of ethnic groups. The degree to which naming practices correspond to individual, ethnic identities may thus offer new, population-wide perspectives upon the progress of everyday cosmopolitanism in an increasingly diverse society.

## Supporting information

S1 Fig Forename ethnicity clusters with WB = White British, WI = White Irish, WO = White Other, IN = Indian, PK = Pakistani, BD = Bangladeshi, CN = Chinese, BA = Black African, BC = Black Caribbean, XX = Any Other. (EPS)

S2 Fig Surname ethnicity clusters with WB = White British, WI = White Irish, WO = White Other, IN = Indian, PK = Pakistani, BD = Bangladeshi, CN = Chinese, BA = Black African, BC = Black Caribbean, XX = Any Other. (EPS)

## References

[1] Cheshire JA. Analysing surnames as geographic data. J Anthropol Sci. 2014; 92: 99–117. 10.4436/JASS.92004 25020015

[2] Darlu P, Bloothooft G, Boattini A, Brouwer L, Brouwer M, Brunet G, et al The family name as socio-cultural feature and genetic metaphor: from concepts to methods. Hum Biol. 2012;84: 169–214. 10.3378/027.084.0205 22708820

[3] Jobling MA. In the name of the father: surnames and genetics. Trends Genet. 2001;17: 353–357. 11377798 10.1016/s0168-9525(01)02284-3

[4] Mateos P. A review of name-based ethnicity classification methods and their potential in population studies. Popul Space Place. 2007;13: 243–263.

[5] Cheshire JA, Longley PA. Identifying spatial concentrations of surnames. Int J Geogr Inf Sci. 2012;26: 309–325

[6] Longley PA, Cheshire JA, Mateos P. Creating a regional geography of Britain through the spatial analysis of surnames. Geoforum. 2011;42: 506–516.

[7] Mateos P, Longley PA, O’Sullivan D. 2011 Ethnicity and Population Structure in Personal Naming Networks. PLoS One. 2011;6: e22943 10.1371/journal.pone.0022943 21909399 PMC3167808

[8] Finch J. Naming Names: Kinship, Individuality and Personal names. Sociology. 2008;42: 709–725.

[9] Lasker GW. Surnames and genetic structure. Cambridge: Cambridge University Press; 1985.

[10] Catney G. Exploring a decade of small area ethnic (de-)segregation in England and Wales. Urban Studies. 2016;53: 1691–1709.

[11] Hicken MT, Kravitz-Wirtz N, Durkee M, Jackson JS. Racial inequalities in health: Framing future research. Soc Sci Med. 2018;199: 11–18. 10.1016/j.socscimed.2017.12.027 29325781 PMC5915332

[12] Petersen J, Longley P, Gibin M, Mateos P, Atkinson P. Names-based classification of accident and emergency department users. Health Place. 2011;17: 1162–1169. 10.1016/j.healthplace.2010.09.010 21646035

[13] Simpson L, Jivraj S, Warren J. The stability of ethnic identity in England and Wales 2001–2011. J R Stat Soc Ser A Stat Soc. 2016;179: 1025–1049. 10.1111/rssa.12175 27773972 PMC5053233

[14] Abramitzky R, Boustan LP, Eriksson K. Cultural assimilation during the age of mass migration. National Bureau of Economic Research, 2016: w22381. https://www.nber.org/papers/w22381

[15] Kandt J, Cheshire JA, Longley PA. Regional surnames and genetic structure in Great Britain. Trans Inst Br Geogr. 2016;41: 554–569. 10.1111/tran.12131 27708455 PMC5032893

[16] Finney N, Simpson L. ‘Sleepwalking to segregation’? Challenging myths about race and migration. Bristol: Policy Press; 2009.

[17] Monasterio L. Surnames and ancestry in Brazil. PloS One. 2017;12: 1–15. 10.1371/journal.pone.0176890 PMC5421764 28481940

[18] Office for National Statistics. Final recommended questions for the 2011 Census in England and Wales: Ethnic group. 2009. https://www.ons.gov.uk/file?uri=/census/2011census/howourcensusworks/howweplannedthe2011census/questionnairedevelopment/finalisingthe2011questionnaire/final-recommended-questions-2011-ethnic-group_tcm77-183998.pdf

[19] Everitt BS, Landau S, Leese M, Stahl D. Cluster Analysis. 5^th^ ed New York: John Wiley & Sons, Ltd; 2011.

[20] O’Brien O, Longley P. Given and Family Names as Global Spatial Data Infrastructure In Longley P, Cheshire J, Singleton A, editors. Consumer Data Research. London: UCL Press; 2018 pp. 53–67.

